# Sodium bicarbonate as a local adjunctive agent for limiting platelet activation, aggregation, and adhesion within cardiovascular therapeutic devices

**DOI:** 10.1007/s11239-023-02852-4

**Published:** 2023-07-11

**Authors:** Kaitlyn R. Ammann, Christine E. Outridge, Yana Roka-Moiia, Sami Muslmani, Jun Ding, Joseph E. Italiano, Elisa Tomat, Scott Corbett, Marvin J. Slepian

**Affiliations:** 1 Department of Medicine, University of Arizona, 1501 N Campbell Ave, Tucson, AZ 85724 USA; 2 Arizona Center for Accelerated Biomedical Innovation, University of Arizona, Tucson, AZ USA; 3 Sarver Heart Center, University of Arizona, 1501 N Campbell Ave, Tucson, AZ 85724 USA; 4 ABIOMED, Inc., Danvers, MA USA; 5 Department of Surgery, Boston Children’s Hospital, Harvard Medical School, Boston, MA USA; 6 Department of Chemistry and Biochemistry, University of Arizona, Tucson, AZ USA; 7 Department of Biomedical Engineering, University of Arizona, 1501 N Campbell Ave, Tucson, AZ 85724 USA

**Keywords:** Thrombosis, Platelets, Sodium bicarbonate, Platelet activation, Platelet aggregation, Platelet adhesion, Blood-contacting devices

## Abstract

Cardiovascular therapeutic devices (CTDs) remain limited by thrombotic adverse events. Current antithrombotic agents limit thrombosis partially, often adding to bleeding. The Impella® blood pump utilizes heparin in 5% dextrose (D5W) as an internal purge to limit thrombosis. While effective, exogenous heparin often complicates overall anticoagulation management, increasing bleeding tendency. Recent clinical studies suggest sodium bicarbonate (bicarb) may be an effective alternative to heparin for local anti-thrombosis. We examined the effect of sodium bicarbonate on human platelet morphology and function to better understand its translational utility. Human platelets were incubated (60:40) with D5W + 25 mEq/L, 50 mEq/L, or 100 mEq/L sodium bicarbonate versus D5W or D5W + Heparin 50 U/mL as controls. pH of platelet-bicarbonate solutions mixtures was measured. Platelet morphology was examined via transmission electron microscopy; activation assessed via P-selectin expression, phosphatidylserine exposure and thrombin generation; and aggregation with TRAP-6, calcium ionophore, ADP and collagen quantified; adhesion to glass measured via fluorescence microscopy. Sodium bicarbonate did not alter platelet morphology but did significantly inhibit activation, aggregation, and adhesion. Phosphatidylserine exposure and thrombin generation were both reduced in a concentration-dependent manner—between 26.6 ± 8.2% (p = 0.01) and 70.7 ± 5.6% (p < 0.0001); and 14.0 ± 6.2% (p = 0.15) and 41.7 ± 6.8% (p = 0.03), respectively, compared to D5W control. Platelet aggregation via all agonists was also reduced, particularly at higher concentrations of bicarb. Platelet adhesion to glass was similarly reduced, between 0.04 ± 0.03% (p = 0.61) and 0.11 ± 0.04% (p = 0.05). Sodium bicarbonate has direct, local, dose-dependent effects limiting platelet activation and adhesion. Our results highlight the potential utility of sodium bicarbonate as a locally acting agent to limit device thrombosis.

## Highlights

- In the local micro-environment, sodium bicarbonate can influence platelet function.
- Sodium bicarbonate led to reduction in platelet phosphatidylserine exposure, thrombin generation rate, agonist-mediated aggregation, and adhesion to glass.
- The effect of sodium bicarbonate on platelet function is concentration dependent. Higher concentrations of sodium bicarbonate lead to more significant effects.
- Sodium bicarbonate may be a potential adjunctive or alternative agent to traditional anti-thrombotic agents.

## Introduction

Current therapy for many forms of advanced cardiovascular disease is increasingly reliant on the use of implantable cardiovascular therapeutic devices [1–4]. While these devices have demonstrated restorative hemodynamic function and efficacy, they remain limited as foreign bodies with significant intrinsic thrombogenicity [5, 6]. Similarly, currently in use are a wide range of anti-thrombotic agents i.e., anti-coagulants and anti-platelet drugs, employed concurrently with devices to mitigate device-related thrombosis [7]. Despite the use of these agents, significant thrombosis-related limitations and adverse events remain, which include device-associated thrombosis, thromboembolic-related complications, and bleeding [8].

A prominent device in widespread use today for cardiovascular hemodynamic support is the catheter-based micro-axial blood pump Impella® (ABIOMED, Danvers, MA). Propelling blood via a high-speed rotating impeller, the Impella® system relies on internal perfusion with a “purge solution” to effectively lubricate high-speed rotating elements, prevent blood ingress, limit protein denaturation and deposition in purge gaps, and limit purge gap thrombosis [9, 10]. Standard purge fluid contains heparin (25–50 U/mL) to directly inhibit thrombin generation and clot formation locally in the purge gaps. While heparin has demonstrated efficacy in reducing purge gap thrombosis and for maintaining purge gap patency, the overall net delivery of heparin to a given patient resulting from purge fluid infusion and exit into the bloodstream may differ due to device variation in the purge gaps [11]. Concurrently with device use, systemic heparin administration is also required to achieve systemic anticoagulation, e.g. for management of acute coronary syndromes. This combination of systemic heparin administration combined with a variable degree of purge fluid heparin delivery further complicates anticoagulation management, potentially increasing bleeding risk [12, 13]. As such, a need exists for an alternative to heparin in purge fluid to reduce risk of variable, additive heparin administration for Impella® patients, thereby simplifying anticoagulation management overall.

Recently, in an attempt to limit excessive use of anticoagulants, associated with bleeding and unpredictable clotting times, clinicians demonstrated that the rate of bleeding and rate of supratherapeutic anticoagulation is lower with sodium bicarbonate added to the purge solution and is effective at ensuring purge patency and limiting device thrombosis, without evidence of systemic consequence [14, 15]. These observations have motivated our group to take a fresh look at the effect of sodium bicarbonate on platelets, and its impact as an adjunctive agent limiting platelet-mediated thrombogenicity.

Sodium bicarbonate is a therapeutic buffering agent often administered to patients with acid/base disorders [16, 17]. Prior studies have shown that sodium bicarbonate is effective as an inhibitor of blood coagulation in vitro and as a means of limiting thrombosis in catheter access locks in vivo [18]. Further, sodium bicarbonate has been shown to exhibit anti-fouling and anti-bacterial properties, limiting protein denaturation, adsorption and biofilm formation on foreign surfaces, contributors to clot formation [19, 20]. As such, this combination of anti-thrombotic and anti-deposition/anti-fouling properties makes sodium bicarbonate an attractive candidate as a purge fluid heparin alternative. Generically, this raises the possibility of sodium bicarbonate acting as a local anti-thrombotic agent, in a defined location or space of a device, yet without significant systemic consequence, as it will be rapidly diluted and buffered with systemic blood exposure. Despite the promising properties and clinical observations related to sodium bicarbonate, limited data exists on the direct effect of bicarbonate on platelet function. In the present study, we hypothesize that sodium bicarbonate, at acceptable pharmacologic levels and reasonable pH for local use, will have a dose-dependent effect in limiting platelet activation, aggregation, and adhesion to non-biological materials. As a first step we examined the effect of sodium bicarbonate on platelet morphology to identify any signs of significant activation via transmission electron microscopy (TEM). Second, we examined the effect of sodium bicarbonate on platelet activation, i.e. platelet P-selectin exposure, phosphatidylserine exposure and thrombin generation rate. Third, we examined the effect of sodium bicarbonate on agonist-mediated platelet aggregation. Finally, we examined the anti-adhesive effects of sodium bicarbonate on fluorescent-labeled platelet adhesion to a non-biological glass surface.

## Methods

### Blood collection and platelet preparation

Human whole blood was obtained via venipuncture from healthy adult volunteers in accordance with University of Arizona IRB-approved protocol (#1810013264). Blood donors provided written informed consent and verbally confirmed to have abstained from caffeine or alcohol consumption prior to blood collection, nor taken non-steroidal anti-inflammatory medications for 2 weeks prior. Blood was collected directly into ACD-A solution (85 mM trisodium citrate, 78 mM citric acid, 111 mM dextrose) to a final concentration of 10% v/v ACD-A in blood.

Platelet-rich plasma (PRP) was obtained via centrifugation of ACD-A anticoagulated whole blood at 300 g for 15 min. Gel-filtered platelets (GFP) were separated from PRP via gel-filtration in Sepharose 2B columns. Platelet counts in PRP and GFP were measured with a Z1 Coulter Particle Counter (Beckman Coulter Inc.). PRP and GFP were diluted to relevant concentrations for experimentation with a modified Tyrode’s buffer solution with 0.1% (w/v) bovine serum albumin (pH 7.4). All active blood components were used for experimentation within 8 h of collection from the human blood donor.

### Purge solution preparation and pH measurement

Purge solutions were prepared in 5% dextrose (w/v) in water (D5W; Baxter International). Sodium bicarbonate (8.4% in water; Baxter International) was diluted in D5W to a final concentration of 25 mEq/L, 50 mEq/L, or 100 mEq/L, as reasonable and consistent with clinical use. Heparin solution (1000 U/mL) prepared with heparin sodium salt (Sigma Aldrich) was diluted in D5W to a final concentration of 50 U/mL. Prior to experimentation, platelet samples (GFP or PRP) were mixed at a 60:40 volumetric ratio with D5W purge solution alone or D5W purge solution formulated with heparin (50 U/mL) or sodium bicarbonate (25, 50, or 100 mEq/L). Platelet-purge solution samples were incubated for 15 min at 37 °C immediately prior to experimentation.

The pH of both sodium bicarbonate and heparin containing purge solutions were measured using a micro-electrode and pH meter (Orion Versa Star Pro; Thermo Scientific) at room temperature. pH measurements were also repeated following 60:40 mixing of the purge solution with either GFP or PRP.

### Platelet morphology: transmission electron microscopy

GFP samples (100,000 platelets/µL) were fixed with 2.5% formaldehyde/2.5% glutaraldehyde mixture in 0.1 M sodium cacodylate buffer (pH 7.4; Electron Microscopy Sciences) for 30 min at room temperature. Fixed platelets were pelleted via centrifugation at 5000*g* × 5 min and pellets were stored in 0.2 M sodium cacodylate buffer (pH 7.4; Electron Microscopy Sciences) at 4 °C until preparation for TEM.

Prepared platelet pellets were fixed with 1% osmium tetroxide/1.5% potassium ferrocyanide for 60 min followed by washing in water and 0.2 M maleate buffer (pH 5.15; Electron Microscopy Sciences) and staining with 1% (v/v) uranyl acetate in maleate buffer for 60 min. Samples were dehydrated via 10-min incubations in increasing concentrations of ethanol (50%, 70, 90%, 100%) and infiltrated in a 1:1 mixture of TAAB resin and propylene oxide overnight at 4 °C. Samples were embedded in TAAB Epon and polymerized at 60 °C for 48 h. Ultrathin 60-nm sections of platelet samples were cut using a microtome (Reichert Ultracut-S; Leica Microsystems) and transferred onto copper grids followed by staining with lead citrate. Sections were examined with a Tecnai G^2^ Spirit BioTWIN transmission electron microscope at an accelerating voltage of 80 kV. Images were captured with an AMT 2 k CCD camera (Hamamatsu ORCA-HR).

### Platelet activation: flow cytometry

Recalcified GFP (20,000 platelets/µL, 1 mM CaCl_2_) were double-stained with fluorescein conjugated anti-CD41-APC (clone MEM-6, Invitrogen) and annexin V-FITC (eBioscience™) or anti-CD42a-FITC (clone GR-P, eBioscience™) and anti-CD62P-APC (clone Psel.KO2.3, eBioscience™) for 30 min at room temperature followed by fixation in filtered 2% (v/v) paraformaldehyde in phosphate-buffered saline (PBS) for 20 min at room temperature. Fixed and stained platelets were then diluted tenfold in PBS prior to analysis. Flow cytometry was conducted utilizing a FACSCanto II flow cytometer (BD Biosciences). Ten thousand events were captured within a defined “platelet” stopping gate established based on their forward versus side scatter characteristics (FSC-A/SSC-A), as compared with standard polystyrene beads of 880 nm and 1350 nm size from the SPHERO™ Nano Fluorescent particle standard kit (Spherotech, Lake Forest, IL). Platelets were identified as CD41 + particles within this gate. Activated platelets, as measured by surface P-selectin (CD62P) expression, were identified as CD62P + platelets and expressed as percentage of CD41 + population. Similarly, platelet phosphatidylserine exposure was measured by bound annexin V and expressed as percentage of CD41 + population. All samples were analyzed in duplicate, and data processed with FCS Express Software (DeNovo Software, V3).

### Platelet activation: thrombin generation rate

Thrombin generation rate, a parameter of platelet activation, was quantified using a modified prothrombinase assay, as previously described [21]. In brief, following incubation with purge solutions, platelet samples were re-calcified (5 mM CaCl_2_) and incubated with acetylated prothrombin (0.2 µM Factor IIa) and 0.1 nM Factor Xa. Chromozyme-TH (0.3 mM; Roche Diagnostics GmbH) was utilized as a chromogenic indicator of thrombin generation in the platelet sample. Absorbance (405 nm), corresponding to thrombin generation, was kinetically recorded in a spectrophotometric microplate reader (VersaMax; Molecular Devices) over a 7-min period. Slopes of the absorbance measurements were recorded as thrombin generation rate.

### Platelet aggregation

Platelet aggregation was measured utilizing a light-transmittance aggregometer (PAP-8E; BioData Corporation). Following incubation with purge solution, PRP samples (100,000 platelets/µL) were re-calcified (1 mM CaCl_2_) and stimulated to aggregate with the addition of 4 separate agonists: 32 µM thrombin receptor activating peptide 6 (TRAP-6; AnaSpec); 12 µM calcium ionophore (A23187); 20 µM adenosine diphosphate (ADP); or 5 µg/mL collagen. Immediately following agonist addition, light transmittance was recorded over a 10-min period. Samples were maintained at 37 °C and stirred at approximately 1000 rpm during aggregation recording. Area under the generated curve was recorded as a measure of platelet aggregation.

### Platelet adhesion: live fluorescent imaging

GFP samples (20,000 platelets/µL; 1 mL) were incubated with 5 µL fluorescent cytoplasmic membrane dye (BioTracker 490; Sigma-Aldrich) for 15 min at room temperature. Immediately following staining, 20 µL of un-fixed GFP were allowed to adhere to clean glass slides for 15 min immediately prior to imaging on an upright microscope (Nikon Eclipse Ni). Adhered platelets (Ex. 484/ Em. 501 nm) were visualized via a 100 × objective oil-immersion lens and captured with CMOS camera (Nikon DS-Qi2). Three images were captured from each sample. Platelet adhesion quantification was performed in MATLAB (Mathworks v.2019a) utilizing the *imbinarize*() and *bwareopen*() functions for precise thresholding and segmentation of adhered fluorescent platelets. Platelet adhesion was calculated as percent coverage per field of view.

### Statistical analysis

Blood donations were taken from a pool of 12 human donors (8 M, 4F; Age 23–32 years). Quantitative experiments were repeated using blood from N ≥ 3 different donors. Assays of each donor sample were performed in duplicate, apart from fluorescent imaging which was performed in triplicate. To account for inter-donor variability in platelet function, purge solutions were run in parallel for each donor and results were quantified as a percent of the D5W negative control. ANOVA with Tukey post hoc test was used to quantify p-values and to identify significant differences between purge solution with additive and D5W solution. All statistical tests were performed at a significance level of 0.05. All values are displayed as mean ± standard error, unless otherwise stated. Data analysis was performed and graphed using GraphPad Prism (v9.0.1).

## Results

To elucidate the influence of sodium bicarbonate on platelet function, human GFP or PRP were incubated with purge solutions containing D5W alone, or D5W with the addition of sodium bicarbonate (25, 50, and 100 mEq/L) of 50 U/mL heparin. Platelet samples were mixed at a consistent 60:40 platelet: purge ratio to mimic the internal Impella® device micro-environment where purge fluid and blood initially mix.

As a first step, we quantified the pH of our purge solution–platelet mixture. Subsequent platelet morphology via transmission electron microscopy (TEM) was qualitatively assessed. Platelet functionality was simultaneously investigated through platelet activation, i.e. platelet thrombin generation rate, P-selectin (CD62P) expression, and phosphatidylserine exposure. Furthermore, platelet aggregability and adhesivity were tested to examine potential alterations in platelet ability to form thrombus in varying purge solution environments.

### pH range of sodium bicarbonate solutions

**Fig. 1:**
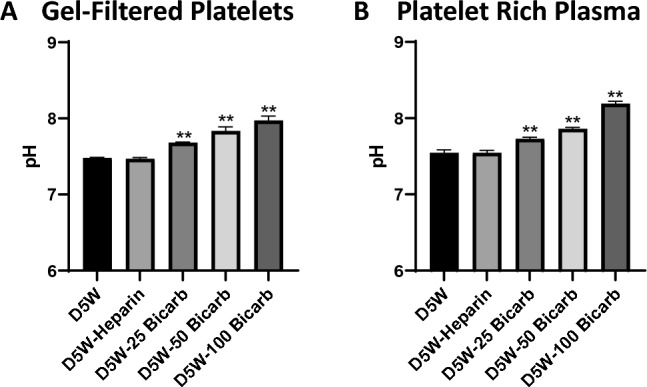
Effect of Sodium Bicarbonate on pH. Human platelets derived from ACD-A anticoagulated whole blood, were incubated (60:40) with purge solution. Purge solutions were formulated in base solution of 5% dextrose in water (“D5W”) with additives 50U/mL heparin (“D5W + Heparin”), 25 mEq/L sodium bicarbonate (“D5W + 25 Bicarb”), 50 mEq/L sodium bicarbonate (“D5W + 50 Bicarb”), or 100 mEq/L sodium bicarbonate (“D5W + 100 Bicarb”). pH was measured after 15 min of incubation with gel-**A** gel-filtered platelets (GFP) or **B** platelet rich plasma (PRP) utilizing micro pH electrode (Orion; PerpHecT ROSS). Values are reported as displayed as mean ± standard error. Data were collected from N ≥ 3 human donors; **indicates p-value < 0.01 in comparison to D5W

The pH of our sodium bicarbonate starting stock solution ranged between 8.60 and 8.68, whereas D5W and heparin solutions had an average pH of 5.83 ± 0.07 and 6.12 ± 0.04, respectively. Following mixing with GFP, the pH of the platelet and D5W mixture was 7.48 ± 0.01 (Fig. 1A). The same mixture with 50 U/mL heparin resulted in a pH of 7.47 ± 0.02. GFP mixture with 25, 50, and 100 mEq/L sodium bicarbonate solution yielded a pH of 7.68 ± 0.01, 7.83 ± 0.06, and 7.97 ± 0.06, respectively. A similar trend was observed following mixing with PRP (Fig. 1B). The pH of PRP and D5W mixture was 7.51 ± 0.01 and the pH of the PRP with heparin was 7.51 ± 0.01. The PRP mixture with 25, 50, and 100 mEq/L sodium bicarbonate solution yielded a pH of 7.71 ± 0.01, 7.85 ± 0.02, and 8.17 ± 0.03, respectively. In total, the operational range to which platelets were exposed in subsequent studies ranged between a pH of 7.47 and 8.17.

### Platelet morphology

**Fig. 2:**
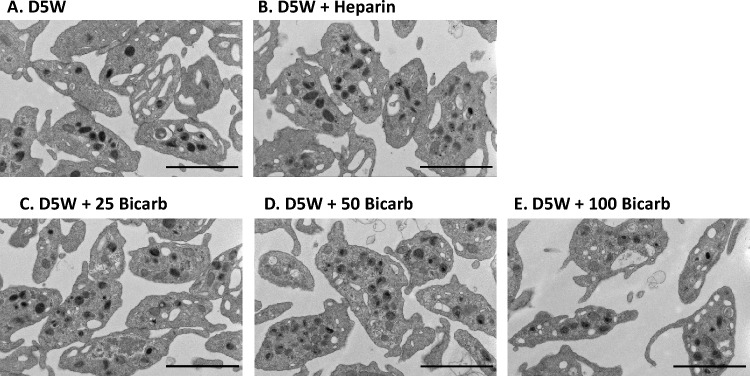
Effect of sodium bicarbonateon platelet morphology. Humangel-filtered platelets (GFP) derived from ACD-A anticoagulated whole blood, were incubated (60:40) with purge solution. Purge solutions were formulated in base solution of 5% dextrose in water (“D5W”) with additives 50 U/mL heparin (“D5W + Heparin”), 25 mEq/L sodium bicarbonate (“D5W + 25Bicarb”), 50 mEq/L sodium bicarbonate (“D5W + 50Bicarb”), or 100 mEq/L sodium bicarbonate (“D5W + 100Bicarb”). Platelets were then pelleted via high-speed centrifugation and prepared for transmission electronmicroscopy (JEOL1200X). Images were captured at 10000× magnification. Scale bars (bottom right) in each image represent 1 μm

TEM was employed to detect significant internal or external morphological alterations of platelets following exposure to various purge solutions (Fig. 2). Platelets exposed to D5W—i.e. a negative control without purge additives, were found to have an overall normal morphology, with an average 2–3 µm diameter and a typical discoid or ellipsoidal shape, in agreement with previous reports of quiescent platelets (Fig. 2A) [22]. For D5W alone, occasional platelets were observed to demonstrate a dilated open canalicular system (OCS), suggestive of mild degranulation, though filopodia, membrane extensions or evidence of overall shape change were minimal. Membrane integrity of platelets within the field of view remained intact, with little to no evidence of overall platelet activation. Addition of heparin or sodium bicarbonate (25, 50, 100 mEq/L) to the D5W purge did not lead to significant alterations in morphology, including OCS dilation (Fig. 2B–E). Notably, no significant differences in platelet morphology between sodium bicarbonate-containing purge solutions and heparin-containing purge solutions were evident. Further, platelets in all purge solutions maintained their membrane integrity with evident microtubular structure proximate to the plasma membrane at high magnification, indicating absence of membrane alteration seen in classically activated platelets.

### Platelet activation

**Fig. 3:**
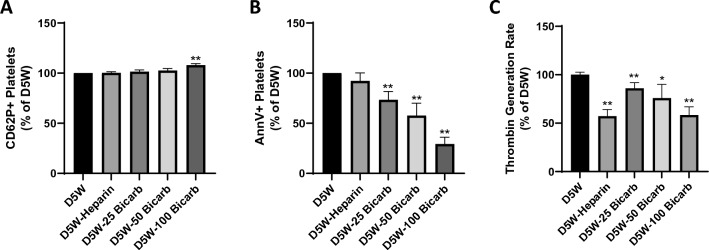
Effect of sodium bicarbonate on platelet activation. Human gel-filtered platelets (GFP) derived from ACD-A anticoagulated whole blood, were incubated (60:40) with purge solution. Purge solutions were formulated in base solution of 5% dextrose in water (“D5W”) with additives 50 U/mL heparin (“D5W-Heparin”), 25 mEq/L sodium bicarbonate (“D5W-25 Bicarb”), 50 mEq/L sodium bicarbonate (“D5W-50 Bicarb”), or 100 mEq/L sodium bicarbonate (“D5W-100 Bicarb”). Flow cytometric detection (FACScantoII, BD BioSciences) of fluorescent platelet activation markers **A** P-selectin (CD62P); and **B** Annexin V binding were measured from the platelet surface. GFP samples were simultaneously measured for **C** thrombin generation rate via colorimetric detection in a modified prothrombinase assay. Values are reported as % of corresponding measurement in D5W solution for each donor, displayed as mean ± standard error. Data were collected from N ≥ 3 human donors, performed in duplicate (n ≥ 6). *Indicates p-value < 0.05; **Indicates p-value < 0.01 in comparison to D5W

As a measure of platelet degranulation, an indicator of platelet activation, flow cytometric detection of P-selectin (CD62P) was measured on the platelet membrane surface (Fig. 3A). Our results indicated no significant difference in average CD62P + platelet populations after addition of 25 mEq/L sodium bicarbonate (p = 0.82), 50 mEq/L sodium bicarbonate (p = 0.61), or heparin (p = 0.99). However, incubation with 100 mEq/L sodium bicarbonate solution led to an average 7.9 ± 1.5% increase in CD62P + platelets, significantly higher than levels seen with D5W alone (p = 0.001) and D5W with heparin (p < 0.01). Sodium bicarbonate at 25 or 50 mEq/L concentrations did not lead to significantly different changes in P-selectin exposure compared to heparin (p > 0.49).

As an alternative to platelet degranulation, phosphatidylserine exposure on the platelet membrane was measured through binding of fluorescent-conjugated Annexin V. In contrast to platelet activation measured by P-selectin exposure, we found an inhibition in phosphatidylserine exposure on platelets after incubation in bicarbonate-containing purge solutions (Fig. 3B). All concentrations of sodium bicarbonate tested led to a significant decrease in platelet phosphatidylserine exposure, with an average decrease of 26.6 ± 8.2% (p = 0.01), 42.3 ± 12.4% (p = 0.01), and 70.7 ± 5.6% (p < 0.0001) for 25, 50, and 100 mEq/L sodium bicarbonate, respectively. The decrease in Annexin V + platelets was positively correlated to the sodium bicarbonate concentration in the purge solution, with 100 mEq/L sodium bicarbonate in D5W exhibiting the most dramatic decrease in Annexin V + platelets. In contrast to sodium bicarbonate, heparin in the purge solution did not lead to a significant difference in the Annexin V + platelet population (p = 0.67). When compared to heparin, sodium bicarbonate had a more significant effect on phosphatidylserine exposure inhibition with an average difference of 18.1 ± 11.5% (p = 0.10), 34.5 ± 14.7% (p = 0.002), 62.9 ± 11.0% (p < 0.005) for 25, 50, and 100 mEq/L sodium bicarbonate, respectively.

As a functional measurement of platelet activation, thrombin generation rate was measured from platelets in various purge solutions (Fig. 3C). Sodium bicarbonate at high concentrations led to a significant decline in platelet thrombin generation rate versus D5W control, quantified as an average decrease of 14.0 ± 6.3% (p = 0.15), 24.2 ± 9.6% (p = 0.05), and 41.7 ± 6.8% (p = 0.03) for 25, 50 and 100 mEq/L sodium bicarbonate solutions, respectively. The decrease in thrombin generation was positively correlated to the sodium bicarbonate concentration in the purge solution, with 100 mEq/L sodium bicarbonate in D5W exhibiting the most dramatic decrease in thrombin generation rate. As expected, heparin, an indirect thrombin inhibitor, also led to a significant decrease (42.9 ± 7.1%; p < 0.001) in thrombin detected in the assay. This was similar to the level of decline exhibited from platelets after exposure to 100 mEq/L sodium bicarbonate purge solution (p = 0.99). Similarly, 25 and 50 mEq/L sodium bicarbonate did not have a significantly different effect on thrombin generation rate as compared to heparin (p > 0.09).

### Platelet aggregation

**Fig. 4:**
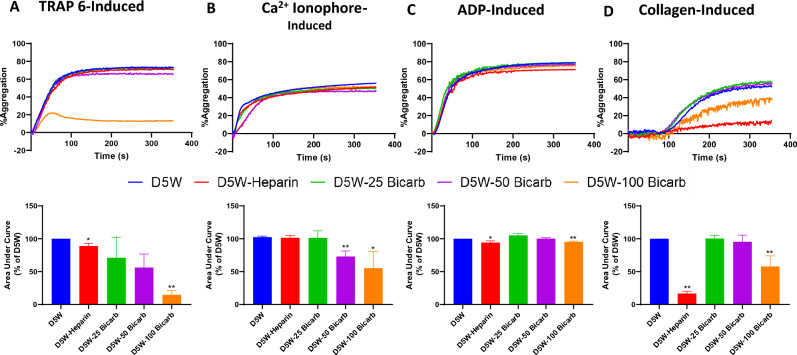
Effect of purge solution on agonist-mediated platelet aggregation. Human platelet rich plasma (PRP), derived from ACD-A anticoagulated whole blood, was incubated (60:40) with purge solution. Purge solutions were formulated in base solution of 5% dextrose in water (“D5W”) with additives 50U/mL heparin (“D5W-Heparin”), 25 mEq/L sodium bicarbonate (“D5W-25Bicarb”), 50 mEq/L sodium bicarbonate(“D5W-50Bicarb”), or 100 mEq/L sodium bicarbonate (“D5W-100Bicarb”). PRP samples were stimulated to aggregate via addition of agonists **A** 32 μM TRAP-6; **B** 10 μM calcium ionophore (A23187); **C** 20 μM ADP; or **D** 5 μg/mL collagen. Aggregation was measured over 10 min with light-transmission aggregometer (PAP-8E; Bio/Data Corporation) traces of aggregation over time are displayed into prow; quantitative data are reported as area under the curve (bottom row). Values are reported as % of aggregation measured in D5W solution for each donor, displayed as mean ± standard error. Data were collected from N ≥ 4 human donors; *Indicates p-value < 0.05;**indicates p-value < 0.01 in comparison to D5W

To evaluate platelet aggregability in the purge solution environment, platelet aggregation was stimulated via addition of defined agonists (Fig. 4). Aggregation via TRAP-6 declined significantly with addition of either sodium bicarbonate or heparin to the purge solution. Addition of sodium bicarbonate led to a decrease in TRAP-6 induced aggregation, with 25, 50, and 100 mEq/L sodium bicarbonate concentrations resulting in an average decrease of 28.9 ± 26.0% (p = 0.78), 43.9 ± 20.6% (p = 0.32), and 85.3 ± 6.6% (p = 0.01), respectively (Fig. 4A). In contrast, addition of heparin led to a significant 11.0 ± 3.4% decline (p = 0.03) in aggregation. Despite the large decline in average aggregation exhibited with sodium bicarbonate solution, there was larger inter-donor variation in the effect of lower sodium bicarbonate concentrations (25 and 50 mEq/L) which led to statistical insignificance. As such, there was no significant difference in TRAP-6 mediated aggregation between platelets incubated in 25 mEq/L sodium bicarbonate versus heparin (p = 0.97) nor between 50 mEq/L sodium bicarbonate versus heparin (p = 0.67). In contrast, platelets in 100 mEq/L sodium bicarbonate led to a more significant decline in aggregation compared to heparin (p = 0.04).

While TRAP-6 stimulates platelet aggregation via PAR1 receptor-binding, calcium ionophore aggregation is receptor independent. Following exposure of platelets to 50 or 100 mEq/L sodium bicarbonate, calcium ionophore-mediated aggregation was significantly decreased (Fig. 4B). Sodium bicarbonate in D5W led to an average decrease in aggregation of 27.0 ± 5.7% (p = 0.04) and 44.8 ± 16.9% (p = 0.03) at concentrations of 50 and 100 mEq/L, respectively. However, low concentration of sodium bicarbonate (25 mEq/L) led to no significant change in aggregation, with an average decline of 1.1 ± 10.2% (p = 0.99). In contrast to TRAP-6-mediated aggregation, platelets exposed to heparin during calcium ionophore-mediated aggregation did not exhibit statistically significant different aggregability, with an average 1.0 ± 3.7% decrease (p = 0.99). Similarly, there was no significant difference in calcium ionophore-induced aggregation between 25 mEq/L sodium bicarbonate and heparin (p = 0.99); however, both 50 mEq/L (p = 0.04) and 100 mEq/L (p = 0.05) sodium bicarbonate had a more statistically significant inhibitory effect on calcium ionophore-mediated aggregation as compared to heparin.

In examining ADP induced aggregation, ADP as an agonist of P2Y1 and P2Y12 receptors, neither sodium bicarbonate nor heparin had a major effect in modulating aggregation (Fig. 4C). Aggregation after platelet incubation in 100 mEq/L sodium bicarbonate led to 3.9 ± 1.0%. decrease (p = 0.21) as compared to D5 alone. Lower concentrations of 25 mEq/L sodium bicarbonate (p = 0.57) and 50 mEq/L sodium bicarbonate (p = 0.99) led to no significant difference in ADP-mediated aggregation as compared to D5W solution alone. Similarly, heparin had no significant effect on ADP-induced aggregation (p = 0.18) with an average decrease of 5.1 ± 2.5% compared to D5W alone. Higher concentrations of 50 mEq/L sodium bicarbonate (p = 0.58) and 100 mEq/L sodium bicarbonate (p = 0.99) had no significant difference in the impact on ADP-induced aggregation as compared to heparin. However, heparin led to an average 10.9 ± 3.9% lower aggregation as compared to 25 mEq/L sodium bicarbonate (p = 0.04).

Collagen-mediated platelet aggregation occurs via binding to platelet surface GPVI or integrins, e.g., α_2_β_1_. Platelet aggregation via collagen was not significantly influenced by 25 mEq/L or 50 mEq/L sodium bicarbonate with an average change of 0.4 ± 4.1% (p = 0.99) and 4.6 ± 6.9% (p = 0.99), respectively. Notably, 100 mEq/L sodium bicarbonate did show a statistically significant decrease in aggregation, corresponding to 42.2 ± 10.8% change (p = 0.001) as compared to D5W alone (Fig. 4D). Heparin led to a larger decline in collagen-induced platelet aggregation, with an average 83.4 ± 3.2% decline (p < 0.0001). Correspondingly, heparin led to significantly lower aggregation compared to sodium bicarbonate with an average difference of 83.7 ± 5.8% (p < 0.0001) vs. 25 mEq/L, 78.7 ± 8.8% (p < 0.0001) vs. 50 mEq/L, and 41.1 ± 12.6% (p = 0.003) vs. 100 mEq/L, respectively.

### Platelet adhesion

**Fig. 5:**
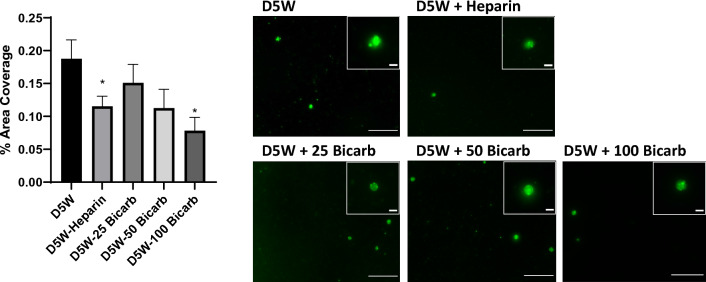
Effect of sodium bicarbonate on platelet adhesion. Human gel-filtered platelets (GFP), derived from ACD-A anticoagulated whole blood, were incubated (60:40) with purge solution. Purge solutions were formulated in base solution of 5% dextrose in water (“D5W”) with additives 50 U/mL heparin (“D5W-Heparin”), 25 mEq/L sodium bicarbonate (“D5W-25 Bicarb”), 50 mEq/L sodium bicarbonate (“D5W-50 Bicarb”), or 100 mEq/L sodium bicarbonate (“D5W-100 Bicarb”). Samples were incubated with cytoplasmic membrane stain (BioTracker490, Invitrogen) and platelets were allowed to adhere to glass microscope slides. Images of slides were taken at 100×  magnification and digitally analyzed for coverage area of platelets adhered to the surface. Data are reported as % area of platelet coverage of the image field of view. Scale bars (bottom right) in each image represent 25 μm. Digitally magnified images of platelet singlets are displayed as insets (upper right) with scale bars representing 3 μm. Values in graph are displayed as mean ± standard error; *Indicates p < 0.05 in comparison to D5W

As an additional assessment of the effect of sodium bicarbonate on platelet functionality in the purge solutions, platelet adhesion to prototypic foreign surface—glass, was quantified (Fig. 5). Lower concentrations of sodium bicarbonate in the purge solution led to statistically non-significant decreases in glass platelet surface coverage, corresponding to average decrease of 0.04 ± 0.03% (p = 061) and 0.08 ± 0.04% (p = 0.22) for 25 mEq/L and 50 mEq/L sodium bicarbonate, respectively. In contrast however, 100 mEq/L sodium bicarbonate in the purge solution led to a significant reduction (0.11 ± 0.04%; p = 0.05) in platelet coverage on the glass surface. Similar to the trends seen in many of the aggregation assays, we found a significant decrease in the platelet coverage in the heparin-containing purge solution (0.07 ± 0.03%; p = 0.04). There were no significant differences in platelet adhesion between heparin and 25 mEq/L (p = 0.61), 50 mEq/L (p = 0.99), or 100 mEq/L (p = 0.15) sodium bicarbonate.

## Discussion

The use of heparin as an antithrombotic agent, while mechanistically effective, adds complexity to clinical anticoagulation management, stemming largely from its intrinsic pharmacokinetic and pharmacodynamic profile and issues related to under- and over-dosing [23, 24]. This issue has particularly been brought to light in the setting of heparin use for concomitant management of a given patient's underlying cardiovascular prothrombotic disease, as well as their therapeutic, but intrinsically thrombotic, device. Recent clinical experience suggests that sodium bicarbonate may be an effective agent to locally reduce thrombosis. In the present study, we explored the possibility of using sodium bicarbonate as a heparin alternative for local use and effect. Here we specifically examined the effect of sodium bicarbonate on platelet morphology and function. Platelet morphology was found to be largely unchanged in the presence of sodium bicarbonate, at the clinically acceptable levels tested, as compared to D5W alone or D5W with heparin. Notably, we observed that sodium bicarbonate exposure led to a reduction of spontaneous platelet activation, as well as to a reduction in agonist-mediated platelet aggregation, particularly at higher concentrations of sodium bicarbonate tested. Additionally, sodium bicarbonate led to a coordinate decrease in platelet adhesion to and coverage of a glass test surface. Overall, our results suggest that in a local environment, such as in a catheter purge gap or in a device intrinsic channel, where bicarbonate concentration is maintained, sodium bicarbonate may offer an environment that decreases platelet thrombogenicity. These observations offer insights into the mechanism of action of sodium bicarbonate, and helps to elucidate operational efficacy, reduced fouling and purge patency when used with the Impella® catheter. These observations also offer translational potential for use in local environments of other devices.

Prior use of sodium bicarbonate as a means of altering platelet function has largely been utilized for preservation and storage of platelets and platelet concentrates [25, 26]. Addition of bicarbonate to stored platelets aids in buffering the pH to maintain it within physiologic range [27]. Low blood pH, or acidosis, is also known to significantly impair coagulation [28]. Intravenous administration of sodium bicarbonate is common therapy for re-neutralizing the pH of the blood and thus can also prevent coagulopathy [17]. Prior literature has demonstrated sodium bicarbonate’s inhibitory effect on the conversion of fibrinogen to fibrin, a necessary step in thrombus formation [29–31]. Wong et al. showed that bicarbonate from both sodium and potassium salts led to an increase of both thrombin clotting time and prothrombin clotting time in vitro as well as in clinical in vivo cases. Other prior studies of sodium bicarbonate have investigated its potential to alter platelet metabolism; however, these studies have shown no direct influence on platelet metabolic activity [25, 26]. Instead, sodium bicarbonate is largely utilized clinically for its indirect effect; its influence on pH and free ionized calcium levels can have significant clinical effects with particular impact on blood coagulation and platelet function when administered intravenously [32, 33].

### Sodium bicarbonate and platelet activation

Platelet activation may be mediated by both biochemical and mechanical means [34, 35]. Central in both of these mechanisms is the role of calcium in activation, as well as in the coagulation cascade [36, 37]. Here, we observe that sodium bicarbonate significantly reduced platelet activation as well as thrombin generation, consistent with effects on both of these processes. While the exact operative biochemical mechanisms of sodium bicarbonate underlying these effects remain undefined, a range of possibilities exist. These include: (1) the alteration of local pH, particularly in a local zone of a device (Fig. 1), subject to limited buffering from other means; (2) intracellular changes related to buffering and carbon dioxide generation; and (3) influence of bicarbonate on calcium availability necessary for platelet function and protein conformation.

Sodium bicarbonate acts as a buffer to preserve physiologic pH and counteract acidity, ultimately influencing metabolic function [11]. In an aqueous environment sodium bicarbonate dissociates to form sodium (Na^+^) and bicarbonate (HCO_3_^−^) ions. Depending on the pH levels in different biological locales, bicarbonate consumes hydrogen ions (H^+^) to form carbonic acid (H_2_CO_3_), that is ultimately converted in the body to water (H_2_O) and carbon dioxide (CO_2_) and excreted via the lungs. In our studies, we examined a reasonable level of sodium bicarbonate as was clinically applicable to the concept here of developing a local anti-thrombotic agent, meaning that the ionic efficacy and pH would be maintained in a privileged region, without it instantly being diluted or buffered by blood. It is understood that if bicarbonate at the amounts used in this study, if added to blood, would be instantly buffered. The concept here is the contained regional effect. As such, it was important to understand the outer boundary of pH as excessive alkalinity in a supraphysiologic level may be caustic. Our goal was to avoid that by operating within a reasonable range (7.47–8.17 pH units). As evidenced by TEM and other data, it did not appear to have significant negative consequences. Prior studies have indicated that bicarbonate does not significantly influence platelet metabolic activity; however, higher alkalinity has been shown to reduce phosphatidylserine exposure in blood cells which coincides with our findings [38].

Another likely mechanism of bicarbonate influence on platelet function is via decrease of free ionized calcium levels [39]. Free ionized calcium (Ca^2+^) is a vital cofactor in platelet activation and the overall coagulation pathway. Additionally, intracellular cytosolic calcium elevation activates multiple platelet signaling events, including those measured in the present study e.g., phosphatidylserine exposure, platelet granule secretion, and thrombin generation. However, the source of calcium Ca^2+^ e.g., intracellular stores or extracellular influx through the plasma membrane, may differ depending on the pathway of activation. In prior work, our group and others have demonstrated that phosphatidylserine exposure relies on a sustained level of cytosolic Ca^2+^, necessitating extracellular influx of calcium into the platelet [34, 37, 40]. Further, phosphatidylserine exposure and thrombin generation are closely linked as phosphatidylserine on the external membrane provides a favorable site for prothrombinase complexes to bind, converting prothrombin to thrombin. As expected, the Annexin V binding and thrombin generation rate quantified in the present study are positively correlated and addition of sodium bicarbonate resulted in similar decreasing trends of platelet activation [41, 42]. From that perspective, adding sodium bicarbonate may function here to reduce levels of free ionized calcium in the local milieu i.e., extracellular Ca^2+^ available for platelet influx. In a similar fashion, ɑ-granule secretion necessary for P-selectin exposure, necessitates elevated cytosolic calcium concentration for cytoskeleton reformation and granule centralization [41]. However, sodium bicarbonate addition to platelets in the present study did not lead to inhibition of P-selectin exposure. Unlike phosphatidylserine exposure, granular secretion, particularly ɑ-granules containing P-selectin, is triggered after release of Ca^2+^ from internal stores [43–45]. Therefore, our results suggest that sodium bicarbonate may influence extracellular Ca^2+^ availability but does not appear to impact internal stores of Ca^2+^ availability required for ɑ-granule secretion and P-selectin exposure. Further investigation is required to fully establish the mechanistic influence of sodium bicarbonate on these different platelet activation pathways and Ca^2+^ availability.

### Sodium bicarbonate and platelet aggregation

Platelet aggregation was also reduced in the presence of sodium bicarbonate. Aggregation can be stimulated by a variety of agonists which bind to specific surface receptors, leading to structural and functional changes of GPIIb/IIIa (integrin ɑ_IIb_ꞵ_3_) on the platelet surface. Activated GPIIb/IIIa can bind fibrinogen, allowing for agonist-stimulated platelets to aggregate [46, 47].

Aggregation agonist TRAP-6 is an analog for thrombin, binding to G protein-coupled receptor PAR-1 on the platelet membrane surface to stimulate aggregation. Collagen interacts with constitutively expressed GPVI on platelets to initiate platelet activation and aggregation. Despite different surface receptors for “outside-in” signaling, both TRAP-6 and collagen lead to an internal signaling cascade within the platelet that ultimately leads to granule secretion, release of Ca^2+^ from internal stores from the platelet dense tubular system, and activation of GPIIb/IIIa necessary for platelet aggregation. After depletion of intra-platelet calcium stores, calcium influx needed for sustained activation is dependent upon extracellular Ca^2+^ availability. As previously mentioned, sodium bicarbonate lowers ionized calcium levels and therefore can abrogate the aggregation process in these cases. This is reflected in the present study in which aggregation was decreased, particularly after addition of high concentrations of sodium bicarbonate.

The mechanism of platelet activation mediated by calcium ionophore, calcimycin (A23187) is receptor-independent. Calcimycin selectively binds to free ionized calcium, allowing transport across the platelet plasma membrane, effectively increasing intracellular Ca^2+^ necessary for platelet activation and aggregation [48]. Our findings of decreased ionophore-mediated aggregation with increasing sodium bicarbonate concentration further supports our results observed with platelet phosphatidylserine exposure, all underpinning the idea that sodium bicarbonate reduces extracellular Ca^2+^ availability for influx into the platelet.

ADP stimulates platelet aggregation through interaction with G protein-coupled receptors P2Y_1_ and P2Y_12_. While ADP in the present study is added exogenously to stimulate aggregation, ADP is also released by activated platelets through δ-granule secretion. While other agonists investigated in the present study showed clear indications of dose-dependent response to sodium bicarbonate, ADP had a less significant response. It is possible that further secretion of ADP from δ-granules in this case created a more robust, sustained aggregation response. Prior work has also demonstrated ADP as an important mediator in calcium signaling between adhered and aggregated platelets and therefore may have further complicated Ca^2+^ influx affected by sodium bicarbonate [49]. Alternatively local buffering effects of sodium bicarbonate may have limited effects on the P2Y1 and P2Y12 purinergic receptors, which are highly integrated into the platelet membrane [50]. Overall, as higher acidity has been shown to impair aggregation and coagulation, it is unlikely that the influence of sodium bicarbonate on increasing alkalinity is the main mechanism at play for reducing agonist-mediated aggregation in the present study [51–53].

### Sodium bicarbonate and platelet adhesion to foreign surface

Glass as a model surface for cell and platelet adhesion is well established and characterized [54–57]. Platelet adhesion to a non-biologic glass surface examined in this study exhibited similar trends as seen with platelet activation by phosphatidylserine exposure. These results were expected as platelet activation typically precedes adhesion to a surface. As such, this is a calcium-dependent process and can similarly be inhibited with reduction in free ionized calcium and is supported by previous reports [54, 58]. Further, phosphatidylserine exposed on the platelet membrane surface is negatively charged, altering electrostatic interaction with the glass surface. Because of evident diminished phosphatidylserine exposed in the presence of bicarbonate, it is possible that the increased negative charge prevented platelet interaction and adhesion to the glass surface.

### Study limitations

Experiments were performed under static conditions. It is possible that non-physiological shear stresses in the high-speed environment of mechanical circulatory support may further influence platelet function and the effect of sodium bicarbonate on the observed parameters. Furthermore, studies were performed with ambient levels of carbon dioxide, below levels observed in vivo. As part of the bicarbonate buffering system, the addition of carbon dioxide could further influence the effect of sodium bicarbonate on pH and local biochemistry observed in the present study. Non-physiological shear and carbon dioxide level as experimental variables were beyond the scope of this project but are a subject of future study.

## Conclusions

Sodium bicarbonate, when used in a local micro-environment and with clinically acceptable dosing, appears to have direct effects on platelet function and coagulation. Under the conditions tested, sodium bicarbonate appears to significantly reduce platelet activation, aggregation, and foreign surface adhesion, with negligible alterations to platelet morphology. This modulation of platelet function occurs in a dose-dependent manner, with more significant changes observed with higher concentrations of sodium bicarbonate. Our results support the utility of sodium bicarbonate as a local antithrombotic agent, for use in geographically defined and constrained regions and spaces, such as in the purge gaps of the Impella®. To be clear, the elevated pH of the bicarb solutions (8.60–8.68 pH) tested limit the practicality of its use for systemic antithrombotic purposes, not to mention whole blood large volume dilution and buffering effects which would extinguish its effects. Operating in a defined space, the effects of sodium bicarbonate appear to be otherwise dominant over other blood clotting effects that would otherwise occur in the larger systemic circulating blood volume. Sodium bicarbonate is a comparatively safe option for use both as an anti-fouling strategy for maintaining device patency, as well as for use in the purge gap microenvironment of the Impella® to limit thrombogenicity. Our results are encouraging and mechanistically supportive of use of sodium bicarbonate as a potential alternative to heparin in as a local anti-thrombotic agent with cardiovascular therapeutic devices.

## Data Availability

Data that support the findings of this study are available for the corresponding author upon request.
